# Ketoacidosis With Canagliflozin Prescribed for Phosphoinositide 3-Kinase Inhibitor–Induced Hyperglycemia: A Case Report

**DOI:** 10.1177/2324709617725351

**Published:** 2017-08-23

**Authors:** Christopher Bowman, Vandana Abramson, Melissa Wellons

**Affiliations:** 1 Vanderbilt University Medical Center, Nashville, TN, USA

**Keywords:** ketoacidosis, drug-induced hyperglycemia, phosphoinositide 3-kinase inhibitor, canagliflozin

## Abstract

*Context*. Many phosphoinositide-3-kinase (PI3K) inhibitors are under trial for cancer treatment. We present a patient taking taselisib who developed ketoacidosis within 1 week of starting canagliflozin. *Case Description*. A 69-year-old female patient with no previous history of diabetes mellitus was enrolled in a clinical trial for taselisib therapy in stage IV breast cancer. Hyperglycemia treatment with metformin was insufficient and not tolerated. The addition of canagliflozin daily resulted in ketoacidosis and hospitalization within 1 week. *Conclusions*. This case report brings together 2 poorly understood and relatively understudied disorders of glucose homeostasis: hyperglycemia due to PI3K inhibition and euglycemic ketoacidosis due to dehydration/SGLT2 inhibition. It demonstrates the complexities of glucose management in the setting of PI3K inhibition. PI3K stimulation (via insulin) in this setting is counterintuitive; therefore, non–insulin-mediated therapies (eg, metformin, thiazolidinediones) might be favored over insulin-mediated therapies.

## Context

Breast cancer is estimated to be the third leading cause of cancer-related death in women.^1^ A commonly mutated pathway in breast cancer (~25%) is the phosphoinositide-3-kinase (PI3K) pathway.^2^ Many PI3K inhibitors are under trial for breast, brain, and lung cancers, as well as hematologic malignancies.^3^ One such agent is taselisib, which is under study for postmenopausal breast cancer. We present a patient taking taselisib who developed ketoacidosis within 1 week of starting canagliflozin.

## Case Presentation

A 69-year-old woman was referred to the endocrine clinic for hyperglycemia. Six weeks prior to her visit, she enrolled in a taselisib (GDC-0032, Genentech) clinical trial that included weekly paclitaxel and dexamethasone 8 mg PO (premedication for paclitaxel), as well as daily taselisib for stage IV breast cancer. Her hemoglobin A1C was 5.4% 9 months prior to her trial start. After trial initiation, her serum glucoses increased from <100 mg/dL (nonfasting, pretrial) to values >270 mg/dL (nonfasting, 17 days after trial initiation). She was prescribed metformin 500 mg twice a day, which was increased to 1000 mg twice a day. However, the patient developed persistent diarrhea not resolved by over-the-counter medicines, and glucoses remained elevated (fasting >150 mg/dL, random >300 mg/dL).

**Figure 1.**
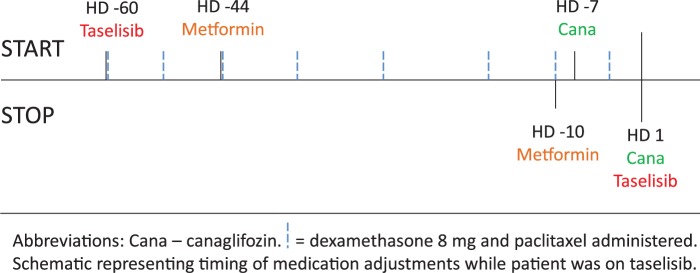
Medication adjustments during taselisib treatment.

A criterion for taselisib clinical trial continued eligibility was maintenance of blood sugars <200 mg/dL. After discussion with the patient, metformin was stopped due to nausea and diarrhea, and canagliflozin 100 mg daily was prescribed (Figure 1). Five days after canagliflozin initiation, the patient presented to oncology for follow-up. At this visit, she reported persistent nausea and diarrhea. Labs at her oncology visit (Table 1; hospital day [HD] −3) included normal electrolytes, creatinine, and CO_2_ (25 mmol/L). The following day she presented to a local emergency room with worsening nausea and vomiting. An abdominal X-ray showed no evidence of bowel obstruction. Her blood urea nitrogen had increased from 11 to 26 mg/dL, and CO_2_ decreased from 25 to 18 mmol/L (Table 1). She received supportive treatment (intravenous fluids, anti-emetics) and was discharged. The following day she returned to the emergency room with nausea, vomiting, and lightheadedness. Her labs were notable for CO_2_ 14, glucose 143, anion gap 21, with normal lactate, moderate acetone (detectable by olfaction by admitting physician when he walked in the room), and arterial blood glass with pH 7.27, pCO_2_ 24 mm Hg, and pO_2_ 105 mm Hg. Urine ketones were positive at 80. Lipase, lactate, aspartate transaminase, alanine transaminase, alkaline phosphatase, and white blood cell count were all within normal limits.

**Table 1.** Laboratory Values and Medicines During Clinic Visits and Hospitalization.

	HD −10	HD −3	HD −1	HD #1	HD #2	HD #4
Na (mmol/L)	136	139	137	139	139	138
K (mmol/L)	3.9	3.7	4.0	4.2	3.0	3.3
Cl (mmol/L)	97	97	101	104	108	105
CO_2_ (mmol/L)	27	25	18	14	21	24
BUN (mg/dL)	12	11	26	28	13	5
Cr (mg/dL)	0.68	0.73	0.79	0.72	0.40	0.39
Glu (mg/dL)	176	151	153	143	105	110
Anion gap	12	17	18	21	10	9
Urine SG		1.028		1.018		
Urine Glu (mg/dL)		>1000		>500		
Urine ketone (mg/dL)		40		80		
Urine blood		Negative		Negative		
Urine LE		Negative		Negative		
Urine nitrite		Negative		Negative		
Pertinent medications	Dex	Dex/Cana				
Stopped medications	Met			Cana; taselisib		

Abbreviations: HD, hospital day (HD #1 = date of admission); Na, sodium; K, potassium; Cl, chloride; CO_2_, carbon dioxide; BUN, blood urea nitrogen; Cr, creatinine; Glu, glucose; SG, specific gravity; LE, leukocyte esterase; Met, metformin; Cana, canagliflozin 100 mg; Dex, dexamethasone 8 mg.

The patient was treated over the next 4 days with intravenous fluids and anti-emetics, while stopping the canagliflozin and taselisib. Her fasting blood glucoses ranged between 75 and 110 mg/dL. Since that hospitalization, the patient has remained off of canagliflozin and has not had any further acidosis.

## Discussion

This case report brings together 2 poorly understood and relatively understudied disorders of glucose homeostasis: (1) hyperglycemia due to PI3K inhibition and (2) euglycemic ketoacidosis due to dehydration/SGLT2 inhibition. The first disorder is a likely common manifestation of a newly emerging class of targeted cancer therapy (ie, hyperglycemia associated with PI3K inhibitors), while the second disorder is an uncommon manifestation of a frequently used oral hypoglycemic medication (ie, euglycemic ketoacidosis with SGLT2 inhibition). This case is thus a good example of the modern complexities of managing the side effects of novel cancer therapies.

The precise mechanism through which PI3K inhibitors cause hyperglycemia is unknown. In healthy individuals, insulin signaling occurs via binding of insulin to an insulin receptor or insulin-like growth factor 1 (IGF-1) receptor, activating insulin receptor substrate proteins, which then bind to PI3K. This binding of PI3K has a variety of downstream effects leading to glucose uptake and glycogen synthesis. Class I PI3K is composed of a heterodimer: p110 (catalytic subunit, consisting of p110α, β, δ, or γ), as well as a p85 regulatory subunit.^4^ This is further divided into IA and IB based on sequence similarity. Inhibition of class IA PI3K via knockout of pik3r1 and pik3r2 genes in mice led to a decrease in insulin secretion and increased glucose intolerance, suggesting that class IA PI3K in β-cells is important for maintaining intracellular Ca^2+^ levels and cell-cell synchronization.^4^

One of the on-target toxicities of taselisib inhibition of class IA PI3K p110α subunit in β-cells is hyperglycemia. In the liver of mice, deletion of p110α reduces insulin sensitivity, impairs glucose tolerance, and increases gluconeogenesis.^4^ In 8-week-old mice, p110α partial inactivation (heterozygous p110α D933A/WT) causes hyperinsulinemia and glucose intolerance.^5^ However, in older mice (66 weeks) with chronic partial p110α inactivation, it was shown that p110α inactivation protects from glucose intolerance, fat accumulation, and age-related reduction in insulin sensitivity compared to control aged mice, suggesting that prolonged PI3K inhibition might not negatively affect organismal metabolism.^6^ In phase I human clinical trials of taselisib, hyperglycemia occurred in up to 38% of patients, with a more common incidence and severity at higher doses.^7^ In phase I trials of alpelisib, another p110α inhibitor, hyperglycemia incidence was 62%.^8^ Hyperglycemia incidence was 30% and 48%, respectively, in phase I trials of the pan-PI3K inhibitors buparlisib and pictilisib.^9^ Hyperglycemia incidence was estimated at 80% in a retrospective study of 4 phase I PI3K inhibitors, specifically 2 targeting pan-PI3K, 1 targeting PIK3α subunit, and 1 targeting PIK3β subunit. These estimates were obtained from clinical trials data from a single cancer hospital (The Royal Marsden Hospital and Institute of Cancer Research, London, UK).^10^

SGLT2 inhibitors appear to cause euglycemic diabetic ketoacidosis (DKA) and ketosis in some patients. A recent case series (9 patients) and an US Food and Drug Administration advisory (73 cases, 44 type 2 diabetes [T2D] cases, 15 type 1 diabetes [T1D] cases) have described euglycemic DKA and ketoacidosis with SGLT2 inhibitors.^11,12^ A phase II double-blinded study of canagliflozin added to insulin therapy in T1D diabetics found that canagliflozin 100 mg and 300 mg was associated with an increased risk of DKA requiring hospitalization as compared with placebo (4.3%, 6%, and 0%, respectively).^13^

Multiple mechanisms for the increased risk for acidosis in patients taking SGLT2 inhibitors are proposed. In T1D, increasing urine glucose excretion with SGLT2 inhibitors results in lower glucose levels. In turn, patients may reflexively lower their insulin dose to prevent hypoglycemia. Less insulin results in a decreased amount of suppression of lipolysis from adipose tissue and more hepatic ketogenesis.^14^ In T2D, SGLT2 inhibitors increase plasma glucagon levels. Elevated glucagon and low relative endogenous insulin production increases lipolysis, releasing free fatty acids from adipose tissue, which are converted to ketone bodies.^15^ Hypovolemia (via osmotic diuresis) also leads to increased cortisol/epinephrine/glucagon, further amplifying insulin resistance, ketogenesis, and lipolysis. This patient had clinical factors that increased her risk of ketoacidosis, specifically hypovolemia and nausea with vomiting. Stopping the canagliflozin at her office visit (HD −3) when her urine showed 40 mg/dL ketones may have prevented her significant ketoacidosis.

Our case demonstrates the complexities of managing the undesired, yet on-target, hyperglycemic effect of acute PI3K inhibition. If PI3K inhibition is the goal, then glucose controlling agents with mechanistic action outside of the PI3K pathway may be preferred. These glucose controlling agents include metformin, thiazolidinediones, acarbose, pramlintide, colesevelam, and SGLT2 inhibitors. However, as our case demonstrates, SGLT2 inhibitors may not be a good therapeutic choice for patients on PI3K inhibitors. It is important to monitor closely for euglycemic ketoacidosis in patients taking SGLT2 inhibitors, especially when nausea, vomiting, or dehydration are suspected. Patients with end-stage cancers may be at increased risk of hypovolemia, making the risk-benefit ratio of SGLT2 inhibitors unsatisfactory. In addition, the use of steroids (eg, dexamethasone, in the case of our patient) may exacerbate hyperglycemia to a degree that an SGLT2 may not be effective. Management of steroid-induced hyperglycemia in combination with PI3K-induced hyperglycemia may be especially difficult. Determining whether this hyperglycemia can be managed without elimination of one or both of these cancer therapies is an area in need of more research. Whether PI3K-stimulating hypoglycemic agents (such as insulin, sulfonylureas, DPP4-inhibitors/GLP-1 agonist, or meglitinides) can be used without risk of tumor growth is another area in need of more research.

## Conclusions

Future studies are needed to establish the prevalence and incidence of drug-induced hyperglycemia in patients administered PI3K inhibitors, as well as the best management approach to ensuing hyperglycemia. Effective hyperglycemia management strategies for patients on PI3K inhibitors might provide the opportunity for patients with known diabetes mellitus to be included in clinical trials of PI3K therapy, and receive a beneficial cancer therapy.
